# DUAL-TASK TRAINING FOR IMPROVING COGNITIVE-MOTOR INTERFERENCE

**DOI:** 10.1093/geroni/igac059.1551

**Published:** 2022-12-20

**Authors:** Tanvi Bhatt, Lakshmi Kannan

**Affiliations:** University of Illinois at Chicago, Chicago,, Illinois, United States; University of Illinois at Chicago, Chicago, Illinois, United States

## Abstract

Dr. Bhatt will discuss the differences in types of cognitive motor interference patterns experienced for different tasks (gaitand volitional versus reactive balance) in cognitively intact versus people with mild cognitive impairment. She will discuss the effectiveness of dual-task training and exergaming on gait, volitional and reactive balance control in older adults with mild cognitive impairment. After 4 weeks of such training, the results showed beneficial effects on improving volitional based performance when performed with a cognitive task (i.e., spatial memory and executive function) and had significant improvement in NIH toolbox (cognitive- increased working memory, episodic memory and executive function, and motor-increased gait speed). However, its positive effects on dual task reactive balance control were limited. Additionally, the speaker will go on to discuss the associations of balance control deficits in mild cognitive impairment with neural correlates (structural and functional brain integrity) to understand the attributing factors to increased fall risk in people with mild cognitive impairment.

